# Vitamin D and Immunity in Infants and Children

**DOI:** 10.3390/nu12051233

**Published:** 2020-04-27

**Authors:** Geneviève Mailhot, John H. White

**Affiliations:** 1 Department of Nutrition, University of Montreal, Montreal, QC H3T 1A8, Canada; 2 Centre Hospitalier Universitaire Sainte-Justine, Montreal, QC H3T 1C5, Canada; 3 Department of Physiology, McGill University, Montreal, QC H3G 1Y6, Canada; 4 Department of Medicine, McGill University, Montreal, QC H4A 3J1, Canada

**Keywords:** vitamin D deficiency, pediatric populations, innate immunity, infectious diseases, autoimmunity, allergies

## Abstract

The last couple of decades have seen an explosion in our interest and understanding of the role of vitamin D in the regulation of immunity. At the molecular level, the hormonal form of vitamin D signals through the nuclear vitamin D receptor (VDR), a ligand-regulated transcription factor. The VDR and vitamin D metabolic enzymes are expressed throughout the innate and adaptive arms of the immune system. The advent of genome-wide approaches to gene expression profiling have led to the identification of numerous VDR-regulated genes implicated in the regulation of innate and adaptive immunity. The molecular data infer that vitamin D signaling should boost innate immunity against pathogens of bacterial or viral origin. Vitamin D signaling also suppresses inflammatory immune responses that underlie autoimmunity and regulate allergic responses. These findings have been bolstered by clinical studies linking vitamin D deficiency to increased rates of infections, autoimmunity, and allergies. Our goals here are to provide an overview of the molecular basis for immune system regulation and to survey the clinical data from pediatric populations, using randomized placebo-controlled trials and meta-analyses where possible, linking vitamin D deficiency to increased rates of infections, autoimmune conditions, and allergies, and addressing the impact of supplementation on these conditions.

## 1. Introduction

Vitamin D is obtained from several sources: The diet, supplements, or seasonal exposure of skin to adequate solar ultraviolet irradiation via photochemical and thermal conversion of the cholesterol precursor 7-dehydrocholesterol. However, many diets are quite poor in vitamin D, and in populous temperate regions, notably in northern Europe and Scandinavia, vitamin D winter, the period where cutaneous vitamin D synthesis cannot occur, is substantially longer than 6 months of the year [1]. Thus, in epidemiological studies, residence at higher latitudes and lower sun exposure have become proxies for risk of a poor vitamin D status. In addition, sun avoidance and conservative dress in more southern regions leads to vitamin D deficiency in several populations worldwide [2]. The risk of vitamin D deficiency occurs at all stages of life, including during pregnancy, in infants and children, as well as in adults. In infants, it can be exacerbated by sun avoidance habits that are usually recommended for very young children, limited body stores, the risk of poor vitamin D content of human milk in exclusively breastfed infants, and a lack of knowledge regarding systematic vitamin D supplementation in some cultures [3].

Vitamin D is best known for its role as a key regulator of calcium homeostasis and bone health in both children and adults [4]. However, it has been the subject of increasing interest in both the scientific literature and the popular press over the last couple of decades, largely because of its “non-classical” actions in tissues unrelated to calcium homeostasis, particularly in the regulation of several aspects of immune system function. Ours goals here are to first provide an overview of the molecular basis for the evidence of a physiological role of vitamin D in tissues unrelated to calcium homeostasis, with a focus on the immune system. We will then detail the clinical evidence for links between vitamin D deficiency and immune system dysfunction in infants and children, with an emphasis on intervention trials, where available. Associations between deficiency and rates of infection; autoimmune conditions, such as type 1 diabetes (T1D) and multiple sclerosis (MS); and allergic conditions, including asthma, will be surveyed. We will also analyze the potential links between vitamin D deficiency and pediatric inflammatory bowel disease and dental caries, both of which are linked to immune system function. Based on the studies detailed below, there is a compelling need for further clinical analysis of the potential benefit of vitamin D supplementation in pediatric populations at risk for several immune-related disorders.

## 2. Overview of the Molecular Basis for the Non-Classical Actions of Vitamin D in the Immune System

The active hormonal form of vitamin D, 1,25-dihydroxyvitamin D (1,25(OH)_2_D_3_), signals through the vitamin D receptor (VDR), a member of the nuclear receptor family of ligand-regulated transcription factors [4]. 1,25(OH)_2_D_3_ is produced by sequential hydroxylations of vitamin D, first by largely hepatic 25-hydroxylation catalyzed by cytochrome P450 2 R1 (CYP2R1) and other enzymes, followed by tightly regulated 1α-hydroxylation by CYP27B1 in peripheral tissues. The classical view of vitamin D action in calcium homeostasis is based on the conversion of 25-hydroxyvitamin D (25OHD), the major circulating form, into 1,25(OH)_2_D_3_ in the kidney. Renal CYP27B1 expression is controlled by key regulators of calcium homeostasis, such as parathyroid hormone and fibroblast growth factor 23 (FGF23) [4]. In the classical model, renal 1,25(OH)_2_D_3_ released into the circulation acts in an endocrine manner to maintain circulating calcium concentrations, most notably by enhancing the intestinal uptake of dietary calcium. Indeed, severe neonatal vitamin D deficiency leads to hypocalcemia and rickets, with defects in skeletal growth associated with softening and weakening of the bones. Descriptions of rickets, along with paleontological evidence, date back millennia [5]. Cod liver oil and sun exposure were proposed as treatments for nutritional rickets as early as the beginning of the 19th century [6]. Unfortunately, 200 years later, nutritional rickets remains a clinical problem and is almost certainly under-reported [7,8]. Moreover, rickets diagnosed in the clinic represents the tip of the iceberg both in terms of the extent of pediatric vitamin D deficiency and because it is often associated with an increased prevalence of diseases unrelated to disrupted calcium homeostasis [5]. These include a number of immune-related disorders, such as increased rates of infections and conditions associated with compromised innate immunity; autoimmune diseases, such as type 1 diabetes (T1D) and multiple sclerosis (MS); and allergic conditions, such as atopic dermatitis [4,9,10]. 

**Figure 1:**
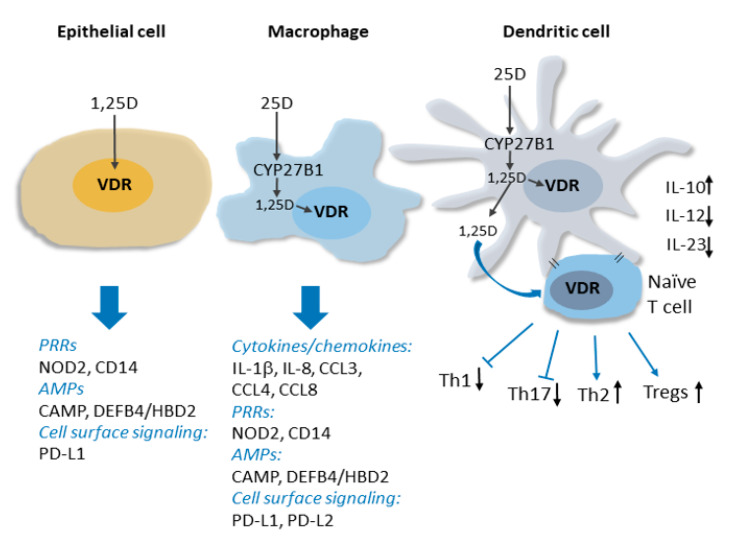
Vitamin D signaling in innate immunity. Intracrine production 1,25D from CYP27B1-catalyzed conversion of circulating 25D in activated macrophages and dendritic cell is shown. The induction of expression by 1,25D signaling through the VDR of genes encoding several types of proteins implicated in innate immune signaling, including cytokines/chemokines, pattern recognition receptors (PRRs) and antimicrobial peptides (AMPs), is indicated. 1,25D signaling within and release from dendritic cells influences dendritic cell maturation and suppresses production of inflammatory Th1 and Th17 cells, favouring Th2 and Tregs. See text for details.

Clinical interest in the links between vitamin D deficiency and these non-classical immune indications has been bolstered by rapidly expanding evidence from laboratory and translational studies that vitamin D signaling is a key regulator of both the innate and adaptive arms of the immune system [3]. The VDR and vitamin D metabolic enzymes are present throughout the immune system, and, importantly, CYP27B1 production in immune cells is induced by pathogen detection and regulated by a complex cytokine network [11,12], and is independent of calcium homeostatic signals. Thus, hormonal 1,25(OH)_2_D_3_ can be produced and act locally in the immune system under conditions of pathogen threat. The discovery of induced CYP27B1 expression in myeloid cells in vitro provides a molecular basis for observations of elevated levels of 1,25(OH)_2_D_3_ produced by macrophages in granulomatous diseases like sarcoidosis, which in extreme cases can lead to hypercalcemia [13] (see Figure 1 for details). 

Innate immune responses are initiated by the detection of pathogen-associated antigens (pathogen-associated molecular patterns; PAMPS) by families of so-called pattern recognition receptors (PRRs). PRR activation and signaling leads to the production of antimicrobial peptides and a network of downstream responses, including an array of cytokines and chemokines, which propagate the signal to other components of the immune system. Studies of 1,25(OH)_2_D_3_-regulated gene expression in innate immune cells have revealed that vitamin D signaling functions upstream and downstream of PRRs to enhance immune responses. This includes inducing the expression of CD14 [14], a cofactor of toll-like receptor 4 PRR signaling, as well as that of NOD2 (nucleotide-binding oligomerization domain protein 2), a PRR defective in a subset of patients with Crohn’s disease [15]. The hormone-bound VDR also directly stimulates the transcription of genes encoding antimicrobial peptides cathelicidin antimicrobial peptide (CAMP/LL37) and human beta/defensin 2 defensin B4 (HBD2/DEFB4) [15,16,17], and readily detectable antibacterial activity is secreted into the media of 1,25(OH)_2_D_3_-treated cells in vitro [16,18]. Importantly, antimicrobial activity in pulmonary surface airway fluid was significantly higher in vitamin D-supplemented patients in a placebo-controlled double-blind randomized trial (RCT) [19]. In the presence of 1,25(OH)_2_D_3_, the expression of several cytokines, including interleukin 1β (IL-1β), a core component of innate immune responses, and the neutrophil chemokine IL-8/CXCL8 are induced, notably in macrophages infected with *Mycobacterium tuberculosis* [20]. Vitamin D signaling also regulates the innate-adaptive immune interface by rendering dendritic cells less inflammatory [4,21,22]. This contributes to suppression by 1,25(OH)_2_D_3_ of peripheral inflammatory T cell responses and enhanced development of T-regulatory (Treg) cells [21,23,24,25]. In addition to the above, genome-wide analyses of vitamin D signaling have revealed that the VDR regulates the transcription of numerous other genes implicated in immune system function [26]. Thus, we are physiologically wired to produce 1,25(OH)_2_D_3_ locally in immune cells in response to pathogens, and vitamin D signaling is a key component of many aspects of immune responses. 

### Antiviral Activity of Vitamin D Signaling: Specific Reference to COVID-19

At this writing, the world is in the grips of the COVID-19 pandemic, which is caused by the SARS-CoV-2 (severe acute respiratory syndrome-Covonavirus-2) virus. As such, along with SARS and MERS (Middle East respiratory syndrome), it represents the third and most severe coronavirus outbreak of this century. Notably, a recent *British Medical Journal* editorial on COVID-19 led to an extended discussion of vitamin D deficiency as a potential risk factor [27]. While COVID-19 is particularly severe in elderly populations, all age groups, including pediatric populations, are susceptible. One study provided evidence that pediatric COVID-19 was associated with coinfections [28], and fears of the spread of SARS-COV-2 in children will grow in many countries with a return to school. Clinical trials have yet to be registered to test the effects of vitamin D supplementation in the prevention/treatment of COVID-19 in children, although they are sure to come. However, clinical evidence is presented below that vitamin D supplementation reduces the rates of respiratory tract infections many of which are viral in nature. There is molecular evidence to support such antiviral activity. The antimicrobial peptide CAMP/LL37, whose expression is strongly inducible by 1,25(OH)_2_D_3_, has antiviral activity against enveloped viruses in vitro and influenza A in vivo [29]. 1,25(OH)_2_D_3_ also enhances the antiviral activity of bronchial epithelial cells in vitro and diminishes rhinovirus replication [30]. While these findings support the notion that hormonal vitamin D induces antiviral activity, it should also be noted that vitamin D signaling acts as a negative regulator of the renin-angiotensin system [31], which includes ACE2 (angiotensin converting enzyme 2), the receptor for SARS-COV-2 ACE2 [32]. ACE2 itself functions as a negative regulator of the renin-angiotensin cascade, and in an animal model, a 1,25(OH)_2_D_3_ analogue enhanced ACE2 expression in vitro [33]. This may not be beneficial in the context of a SARS-COV-2 infection; it has been hypothesized that patients being treated with ACE inhibitors for hypertension, which enhance ACE2 expression, may be at an increased risk for the development of severe COVID-19 [34].

## 3. Vitamin D and Infectious Diseases in Pediatric Populations

As developed above, there is extensive molecular evidence supporting vitamin D supplementation of deficient populations as a means to combat both the incidence and severity of infectious diseases. This would not be of substantial clinical relevance if populations were generally vitamin D sufficient. However, observations of widespread vitamin D deficiency [2] suggest that supplementation would be of clinical benefit. Notably, a survey of 1006 adolescents in 10 cities in 9 geographically dispersed European countries found that 80% of the subjects had 25OHD levels of less than 75 nM, considered the threshold of sufficiency, and that ~42% were either deficient (27.5–49.99 nM; 27%) or severely deficient (<27.5 nM; 15%) [35]. Moreover, poor vitamin D status in adolescents can be exacerbated during vitamin D winter and by high body mass index (BMI) [36]. The observations of widespread deficiency in European adolescents is consistent with estimated dietary intakes, which were found to be deficient in vitamin D [37]. They are also in line with the general European population; an analysis of 14 population studies in 55,844 European individuals concluded that 40% had 25OHD levels below 50 nM and that poor vitamin D status was elevated in dark-skinned subgroups [38]. This has led to calls for the measurement of 25OHD levels in risk populations, including pregnant women, young children, and adolescents, using standardized protocols [39]. 

Poor vitamin D status is also rampant in more southerly countries, such as India, where it ranged from ~40%–70% in pregnant women and infants to over 80% in school-aged children [40]. In addition, it is a predictor of infections in Indian children [41]. In a Yemeni study, a diagnosis of rickets was a predictor of a poor response to therapy in children hospitalized with severe pneumonia [42]. Strong support for the benefits of vitamin D supplementation to reduce the risk of infections came from meta-analyses of RCTs by Martineau and colleagues, which revealed that daily or weekly vitamin D supplementation, but not bolus dosing, reduced the rates of upper respiratory tract infections (URTIs). Moreover, the effect was most pronounced in subjects who were the most vitamin D deficient [43,44]. In a subgroup analysis (8 studies), vitamin D supplementation reduced the risk of URTIs in a mixed population of 513 children (1.1–15.9 years; adjusted OR: 0.60; 95%-CI 0.46 to 0.77). One of these studies was an RCT in Japanese schoolchildren [45], which provided evidence that vitamin D supplementation reduced the rates of seasonal influenza A. The results were most striking in the sub-group who had not previously received supplements. Similarly, in an RCT using cluster randomization, classrooms of 247 Mongolian children were randomly assigned during winter to daily supplementation with unfortified regular milk or milk fortified with vitamin D_3_ [46]. Supplementation halved the risk of parent-reported acute respiratory infections. This study was remarkable for several reasons: Notably, median serum 25OHD levels were very low at baseline (7 ng/mL; 17.5 nM), and the daily vitamin D dose used was low (300 IU/d), although supplementation raised the median 25OHD levels to 19 ng/mL (47.5 nM). Another RCT evaluated whether vitamin D supplementation was effective in reducing the number of cases of episodes of recurrent acute otitis media (AOM) in 116 otitis-prone children [47]. Subjects received daily doses of 1000 IU of vitamin D daily or placebo over a 4-month period, which led to a significant reduction in the number of children suffering a single AOM episode (26/58 vs. 38/58; *p* < 0.03). In contrast, an RCT using bolus dosing (100,000IU every 3 months for 18 months) as an approach to reduce the risk of pneumonia in children in Kabul, Afghanistan did not show any benefit of supplementation [48]. In a related RCT, no benefit was observed when a single dose of 100,000IU was used along with antibiotics to reduce the recurrence of pneumonia in children [49]. Similarly, 300,000IU bolus doses in children under five had no significant beneficial effect on the prevention of recurrent pneumonia [50]. 

A recent systematic review and meta-analysis of 7434 pediatric patients investigated the association of vitamin D status with mortality in children with acute or critical conditions [51]. The study found high levels of 25OHD deficiency (47% < 50 nM) in the group as a whole and higher levels (64%) in children with sepsis. Moreover, vitamin D deficiency was associated with increased mortality. These findings were consistent with those from a Canadian study, which did not find a difference in serum 25OHD levels in children with acute lower respiratory tract infections and controls but did see more frequent vitamin D deficiency in children admitted to intensive care with severe disease [52]. Collectively, the above results are striking because they support the hypothesis that the maintenance of vitamin D sufficiency should reduce rates of infections in pediatric populations. They are also striking because of the apparent lack of efficacy of bolus dosing. Bolus dosing is practical from a clinical perspective because it eliminates concerns about compliance, and, given the relatively long half-life of circulating 25OHD, should ensure that serum 25OHD remains above the level of sufficiency for several weeks. It can be speculated that daily or weekly dosing may lead to elevated levels of intracellular vitamin D, perhaps stored in lipid droplets, which may serve as a source of local production.

## 4. Crohn’s Disease

Crohn’s disease (CD), whose incidence is rising [53], is a relapsing-recurring inflammatory bowel condition resulting from defective intestinal innate immune homeostasis [54]. CD is notable because its incidence tends to rise with increasing latitude and for its association with low sun exposure early in life [55,56]. Although about one quarter of cases are diagnosed during childhood, pediatric CD is less well studied than adult disease. This is unfortunate as a cohort study identified 404 pediatric CD patients (0-17 years at diagnosis) between 1998 and 2002 in a population base of 1,312,141 in northern France. Disease complications occurred in a high percentage of these patients and 34% required surgery during the 5 years after diagnosis [57]. Based on the studies detailed below, there is compelling evidence that vitamin D supplementation may be of therapeutic benefit in pediatric Crohn’s patients [58].

There is a strong molecular and genetic basis for a potential therapeutic role of vitamin D signaling control of CD. The hormone-bound VDR regulates the expression of multiple human CD susceptibility loci, including the pattern recognition receptor *NOD2*, also known as *IBD1* (inflammatory bowel disease 1) [15,59]. The VDR also directly induces expression of gene encoding the cell surface glycoprotein programmed cell death ligand 1 (PD-L1/B7-H1/CD274) [60], which interacts with PD1 on the surface of T cells to suppress T cell-mediated inflammatory responses. In a mouse model, defective intestinal epithelial Pd-l1 expression led to gut inflammation, underlining the key role of PD-L1 in suppressing pathological inflammation in the periphery [61]. Genome-wide association studies have revealed that several CD susceptibility loci encode proteins active in innate immune signaling and autophagy (which is critical for the control of intracellular pathogens) [54], both of which are stimulated by vitamin D signaling [26,58].

Several clinical studies, including generally small intervention trials, have provided evidence that vitamin D supplementation reduces disease activity [58]. While studies with children are limited, one examining dosing optimization of pediatric CD patients found that inflammatory markers were lower with high-dose vitamin D treatment (31 children receiving 1000 IU daily in summer and 2000 IU in winter and spring) [62]. The findings of recent meta-analyses of observational studies and RCTs [63,64] collectively confirm that a poor vitamin D status is associated with increased disease activity and that supplementation lowers it. However, based on subgroup analysis, the study of RCTs noted it was too early to make the same conclusions for pediatric CD patients [63]. It would therefore be worthwhile to conduct an RCT examining the effects of robust vitamin D supplementation on disease activity in a pediatric population. In this regard, there is an ongoing RCT at eight Canadian institutions looking at the impact of vitamin D supplementation of newly diagnosed children with CD on the rate of CD relapse (Clinicaltrials.gov: NCT03999580; unpublished clinical trials cited are listed in Table 1).

Although the above results provide support for the therapeutic benefit of vitamin D supplementation in CD, the efficacy of oral supplementation may be complicated by intestinal malabsorption, particularly in the case of patients who have undergone bowel resections and ostomy procedures. In one study, two patients who had recently undergone bowel resections and ileostomies, one of which had CD, failed to respond to aggressive oral vitamin D_3_ supplementation [65]. However, the patients did respond to high-dose sublingual vitamin D_2_ treatment. An alternative in such cases is moderate UVB exposure. Notably, in a CD patient with malabsorption due to multiple bowel resections, exposure to a series of non-erythemic doses of UVB on a tanning bed dramatically improved her vitamin D status [66]. 

## 5. Vitamin D Deficiency and Dental Caries

Dental caries (cavities) arise through the breakdown of teeth by acidic secretions from bacteria in dental plaque. Numerous studies have linked vitamin D deficiency during pregnancy and infancy to an increased rate of early childhood caries [67,68,69]. An association between vitamin D deficiency and dental caries was brought to the forefront by a systematic review and meta-analysis published in 2013 [70]. It surveyed 24 trials that included 2827 children, which generated a pooled relative rate estimate of 0.53 (95%-CI, 0.43–0.65) for the effect of vitamin D supplementation. There was substantial heterogeneity of the results between trials and some evidence of publication bias, generating a low-certainty conclusion when all trials were included. However, when the review was limited to higher-quality studies, it reduced the heterogeneity between trials and provided evidence that vitamin D was highly efficacious. The more restrictive analysis increased the estimated reduction in caries from 47% to 54% [70].

Vitamin D sufficiency may protect against caries through its role in tooth development [71,72], and molar-incisor mineralization [73]. However, vitamin D sufficiency may also be protective through its capacity to boost antimicrobial innate immunity and suppress inflammation. An inverse correlation was observed in 6700 adults between gingival inflammation and serum 25OHD levels [74]. A Swedish study of children found a correlation between poor vitamin D status and increased risk of caries. Notably, vitamin D status in this study correlated positively with levels of the 1,25(OH)_2_D_3_-inducible antimicrobial peptide CAMP (LL37) in saliva [75]. CAMP/LL37 is efficacious against major bacterial species found in plaque, such as *Streptococcus mutans* [76]. In a recent RCT, vitamin D supplementation enhanced the levels of antimicrobial activity in lung surface airway fluid [19]. This suggests that enhanced secreted antimicrobial activity should be detectable in an RCT of vitamin D supplementation and is consistent with the induced secretion of antimicrobial activity by 1,25(OH)_2_D_3_-treated cells in vitro [16]. Given these results, it would be of interest to conduct an RCT to determine whether vitamin D supplementation enhances antimicrobial activity in saliva against major bacterial components of dental plaque.

## 6. Links between Vitamin D Deficiency and Autoimmune Conditions in Pediatric Populations

There is extensive preclinical and clinical literature on the links between vitamin D deficiency and autoimmune conditions, in particular type 1 diabetes (T1D) and multiple sclerosis (MS). The effects of 1,25(OH)_2_D_3_ on T cell function are likely key to its potential capacity to limit autoimmunity. Expression of the VDR in T lymphocytes was discovered in the 1980s, and 1,25(OH)_2_D_3_ attenuates T cell activation and proliferation [77,78], regulating the function of several T cell subsets [26]. Briefly, vitamin D suppresses T cell-driven inflammation while enhancing the action of suppressive Treg cells. Vitamin D signaling in dendritic cells alters their metabolic profile, producing a more tolerogenic phenotype and enhancing interleukin-10 secretion, which promotes Treg production [26,79,80]. There is also support for the potential of vitamin D supplementation in attenuating autoimmunity from studies in mouse models of human autoimmune diseases [81]. At present, clinical use is limited to psoriasis, which is treated with topical application of vitamin D or its analogues [82]. However, overall, there is no consensus for the therapeutic benefit of vitamin D supplementation in the treatment of autoimmune conditions, and, unfortunately, many studies are limited by group sizes.

### 6.1. Juvenile-Onset Type 1 Diabetes

The incidence of T1D, a multifactorial autoimmune condition leading to destruction of the insulin-producing β-cells of the pancreas, is increasing worldwide [83]. There is intriguing preclinical evidence supporting a preventive role of vitamin D, most of which has been generated in the non-obese diabetic (NOD) mouse model of human T1D [84]. In work done by the Mathieu group with the NOD model, vitamin D deficiency in early life increased the incidence and accelerated onset of the disease [85]. Moreover, 1,25(OH)_2_D_3_ and its analogues are more efficacious at preventing insulitis and diabetes in NOD mice when administered early, prior to the onset of immune-mediated beta cell attack [86,87,88], and long term, high-dose 1,25(OH)_2_D_3_ treatment reduced the incidence of diabetes in NOD mice [88]. Intriguingly, immune-mediated induction of CYP27B1 expression is attenuated in NOD mice [89].

Globally, clinical studies of vitamin D supplementation in all age groups with existing T1D are inconclusive but suggest that early intervention may preserve the remaining pancreatic islet β-cell function [81]. In one intervention trial, a protective effect of 1,25(OH)_2_D_3_ supplementation in preserving residual β-cell function in adult-onset disease only occurred when the duration of disease was less than 1 year [90]. There is some evidence for the seasonal onset of T1D in both adults and children, coinciding with low circulating 25OHD levels [91,92], which would be consistent with a poor vitamin D status accelerating disease onset. Nonetheless, evidence for a role of vitamin D deficiency in contributing to T1D incidence remains mixed. For example, two studies did not find any correlation between 25OHD levels measured at birth or in infancy and the risk of developing T1D during childhood [93,94]. Several case-control studies examining the relationship between serum 25OHD levels or vitamin D intake during pregnancy and the risk of T1D in offspring also provided mixed results [83]. However, earlier meta-analyses of observational studies supported the notion that supplementation early in life may contribute to T1D prevention [95,96]. 

Although no RCTs were found examining the potential efficacy of vitamin D supplementation in the prevention of juvenile-onset T1D, case-control studies do provide some evidence for the benefit of vitamin D either as a supplement or in food intake. Of these, two are notable because of their size. A large European-based case-control study screening for early risk factors for the development of T1D concluded that vitamin D supplementation during infancy was beneficial [97]. Similarly, vitamin D supplementation during the first year of life correlated with a reduced incidence of T1D in a Finnish birth-cohort study, and the degree of benefit was greater for infants supplemented with 2000IU/day relative to a lower dose [98]. A Norwegian study (545 cases, 1668 controls) suggested that frequent supplementation (five times or more a week) with cod liver oil in the first year of life lowered the risk of development of T1D [99]. Remarkably, however, this study did not find any evidence that vitamin D supplementation in other forms, even frequent, provided any benefit. Overall, these findings, although inconclusive, are compelling, and strongly suggest that an RCT examining a preventive role of vitamin D supplementation in T1D, at a minimum in the first year of life, would be in order. A robust dose, perhaps as high as 2000 IU/day should be used in the treatment wing. However, this leaves the field in a bit of a bind as the duration of follow-up of such a study should likely be at least 10 years. Nonetheless, the efficacy of vitamin D and the timing of its supplementation will be important issues to resolve in order to reach a consensus. Notably, a recent position paper from multiple German clinical/nutrition societies concluded that “Based on currently available studies, routine vitamin D supplementation is not recommended for children aged >2 years, even when they have serum concentrations below reference values” [100].

### 6.2. Multiple Sclerosis

MS is characterized by an autoimmune attack on the protective myelin sheath covering nerve fibers. It is perhaps the autoimmune condition with the strongest association between vitamin D deficiency and the risk of development. MS risk is multifactorial and has parallels with Crohn’s disease, as discussed above, in that there is an inverse correlation between disease incidence and previous sun exposure. For example, one case-control study found that higher sun exposure in 6- to 15-year-old children reduced the risk of subsequent development of MS [101]. Moreover, also similar to CD, an increased incidence of MS has been linked historically to higher latitudes [102]. However, one systematic review suggested that MS incidence has been rising at lower latitudes since 1980 [103]. Moreover, pure geographical interpretations of the risk of MS development are complicated by ethnicity, e.g., risk is low in native Siberians and populations in northern Scandinavia but high in Palestinians, indicative of genetic factors [102,104]. A number of cohort studies have provided evidence that vitamin D deficiency contributes to the risk of pediatric MS [105], and another study established a correlation between serum 25OHD levels and relapse rates [106]. Adolescent obesity is also associated with risk of the development of MS, although here it is not clear whether adiposity per se is a risk factor or whether it is associated with the sequestration of vitamin D metabolites and lower circulation of 25OHD levels [107].

Further strong evidence that vitamin D deficiency is a key risk factor for MS has been provided by Mendelian randomization (MR) studies. An MR study uses known functional variants of genes to probe the causal effect of a specific variable on disease occurrence, severity, or outcome. Four single nucleotide polymorphisms (SNPs) were identified in genes encoding enzymes/binding proteins that influenced circulating 25OHD levels: *CYP2R1*; *CYP24A1*, which encodes the enzyme that initiates the catabolism of 25OHD and 1,25(OH)_2_D_3_; *GC*, encoding the major serum 25OHD binding/carrier protein; and *DHCR7*, which encodes the enzyme that converts 7-dehydrocholesterol to cholesterol [108]. As these are genetic variants, their individual and collective impacts on disease represent causal effects of life-long vitamin D deficiency. Remarkably, the use of these variants in a large-scale MR study of MS (14,498 cases and 24,091 controls) provided evidence that genetic variants lowering circulating 25OHD levels strongly enhanced susceptibility to the development of disease [108]. Moreover, poor vitamin D status may influence the age of disease onset. Symptoms of MS usually first appear in young adulthood (20–40 years of age), and pediatric-onset disease accounts for only approximately 5% of all cases. Remarkably, another MR study of 569 pediatric MS cases in Sweden and the US (Sweden 175 cases, 5376 controls; US, 394 cases, 10,875 controls; 16,820 total) found that increased circulating 25OHD was associated with decreased odds of developing pediatric MS (OR 0.72, 95%-CI 0.55–0.94; *p* = 0.02) [109]. The results were striking, as the SNPs examined accounted for only 2.8% of the variance in circulating 25OHD levels. Collectively, the results above are compelling and suggest that poor vitamin D status is a key contributor to the risk of development of MS and may be a contributing factor to pediatric onset. 

### 6.3. Other Autoimmune Conditions

Clinical studies testing the association of vitamin D status with other juvenile-onset autoimmune conditions are more limited. Systemic lupus erythematosus (SLE), which is more common in females than in males, is characterized by elevated levels of autoantibodies to self-nucleic acids or nucleoproteins [110]. Clinically, inflammation in SLE may affect multiple organs (joints, kidneys, skin, heart, lungs, vasculature, the nervous system, etc.). There is one RCT examining the efficacy of vitamin D supplementation in adolescents with SLE, which provided evidence that vitamin D reduced disease activity and fatigue scores [111]. The trial is notable for the robust vitamin D dose used (50,000 IU/week for 24 weeks), which boosted serum 25OHD levels from 19.3 to 31.3 ng/mL (48.3–78.3 nM). This result is encouraging, and there is some support for it from RCTs and other studies in adult populations. An inverse correlation has been established between serum 25OHD levels and SLE disease activity in adult patients [112]. In an RCT in SLE patients, supplementation with 2000 IU/day of vitamin D raised serum 25OHD levels from 19.8–28.7 ng/mL (49.5–71.8 nM) and significantly improved the levels of inflammatory and hemostatic markers [113]. Globally, however, findings are not unanimous. For example, in another RCT with daily doses of 2000 or 4000 IU/day, supplementation did not diminish the interferon signature associated with the disease [114].

Juvenile idiopathic arthritis (JIA) is a collective term for a heterogeneous group of arthritis-like diseases of unknown origin [115]. Vitamin D deficiency has been considered as a potential environmental determinant of JIA [116], and an Australian case-control study established a link between higher life-long sun exposure and reduced risk of JIA [117]. At this stage, clinical data is best described as “emerging”. A systematic review published in 2013 found, unsurprisingly, that rates of vitamin D deficiency were high in a number of studies of JIA patients but also highlighted the lack of hard data linking vitamin D status or supplementation to disease activity [118]. One study found extensive vitamin D deficiency but did not find a correlation between vitamin D status and disease activity in a cohort of patients (mean age 10.6 years) with JIA [119], whereas studies of small Turkish and much larger German cohorts did find such an association [120,121]. A small RCT was recently published examining the effects of supplementation of JIA patients in China with 2000 IU/day of vitamin D (20 patients in treatment group, 22 in control group) on disease activity and bone parameters [122]. While supplementation raised serum 25OHD levels, no effects were observed on the disease activity score or on bone mineral density over the 24-week period of the trial. Hopefully, more and larger trials of this kind for JIA will follow and establish whether vitamin D supplementation may be of therapeutic benefit for these patients.

## 7. Allergic Conditions

Similar to other immune conditions, rising trends in the incidence of allergic diseases have coincided with the increase in the prevalence of a vitamin D deficient/insufficient status reported worldwide. Epidemiological studies have shown an association between the risk of allergic conditions and latitudes, ultraviolet radiation (UVR) intensity, and birth season. In general, a greater risk of allergy is observed in individuals living furthest from the equator, residing in low-UVR intensity areas, and in those born during winter months [123,124,125]. The emerging importance of vitamin D in prenatal and early postnatal development of the immune system is suggested to play a role in the association between vitamin D status and the onset of allergic disorders. This view is, however, challenged by the so-called “vitamin D allergy hypothesis”, which attributes the increased incidence of allergic diseases to the immunological side effects of vitamin D supplementation initiated during infancy [126]. Other studies have supported the association between infant vitamin D supplementation and the risk of atopic diseases in childhood [127] and adulthood [128]. Moreover, maternal levels of 25OHD >75 nM in the third trimester of pregnancy have been linked to increased odds of developing visible eczema in children at 9 months and having asthma at 9 years, although the number of cases was small and loss to follow-up high, particularly at 9 years [129]. Potential explanations to reconcile these conflicting results relate to the U-shaped association found between cord blood 25OHD levels and risk of allergic diseases in early childhood [130] and between 25OHD levels and immunoglobulin E (IgE) levels in adults [131]. It is speculated that the balance between the systemic T-helper 2 (Th2)-skewing effect of vitamin D and its local tissue anti-inflammatory effects may vary depending on the circulating concentration of 25OHD [124]. 

### 7.1. Asthma

Asthma is a common chronic inflammatory disorder that primarily affects the respiratory system. It is a very heterogeneous condition comprising many phenotypes that differ depending on the nature of the trigger (e.g., allergic vs. non-allergic), the age of onset (preschoolers vs. schoolchildren vs. late onset), the pathophysiology involved (high vs. low Th2-mediated responses, eosinophilic vs. non-eosinophilic), and whether asthma symptoms are transient or persist into adulthood [132]. Its clinical presentation is also variable with the occurrence of asthma-like symptoms (e.g., prolonged coughing, recurrent wheezing) in preschoolers that may resolve with time or transition to more severe symptoms, leading to irreversible obstructive changes in the lungs.

The use of vitamin D in the primary prevention of asthma is an area of active investigation and stems from preclinical studies, mostly supporting the involvement of vitamin D in lung growth and the development of the immune system [133,134,135]. A decreased risk of childhood asthma (RR 0.87, 95%CI 0.75–1.02) in mothers with maternal or cord blood 25OHD levels in the highest vs. lowest category was demonstrated in a meta-analysis of 15 prospective studies with 12,758 participants and 1795 asthma cases [136]. Interestingly, when data were stratified according to the timing of 25OHD levels’ measurement (i.e., early-mid vs. late pregnancy), the association reached significance only when levels were assessed during early pregnancy (RR 0.73, 95%CI 0.58–0.92). These results were corroborated in a subsequent meta-analysis showing a similar trend between maternal 25OHD levels and the risk of asthma in children ≤ 5 years (OR 0.81, 95%CI 0.65–1.01; 27,776 participants pooled from 6 cohort studies) [137]. In line with these data, a recent case-cohort Danish study reported that children with birth levels of 25OHD in the highest quintile had a lower risk of having asthma between the ages 3 and 9 (fully adjusted HR 0.51, 95%-CI 0.35–0.75) [138]. Since the lung and immune system develop early in gestation but continue their development postnatally, these data indirectly provide evidence for the importance of vitamin D at these stages of development and the consequences that prenatal/postnatal vitamin D insufficiency may have on child health. As such, two large trials of vitamin D supplementation during pregnancy were initiated to determine the impact of prenatal vitamin D on the risk of asthma/recurrent wheezing in children. The Vitamin D Antenatal Asthma Reduction Trial (VDAART) enrolled 806 pregnant women between 10 and 18 weeks of gestation from three sites across the United States. Eligibility criteria included a history of allergic diseases in the mother or in the biologic father. The intervention arm consisted in the administration of daily 4000 IU vitamin D_3_ combined with a multivitamin containing 400 IU D_3_ until delivery. The placebo group received a matching placebo tablet plus the multivitamin with 400 IU D_3_ [139,140]. Using a very similar design, the Copenhagen Prospective Studies on Asthma in Childhood 2010 (COPSAC_2010_) recruited 581 Danish women between 22 and 26 weeks of gestation. Participants were randomized to receive either 2400 IU vitamin D_3_ combined with a multivitamin containing 400 IU D_3_ or only the multivitamin until delivery [141]. The combined analysis of these two trials revealed a significant effect of vitamin D intervention on reducing the risk of developing asthma/recurrent wheeze in the first three years of life (OR 0.74, 95%-CI 0.57–0.96). Unexpectedly, the effect was driven by women with baseline serum 25OHD levels ≥ 75 nM (OR 0.54, 95%-CI 0.33–0.88) vs. women with baseline 25OHD levels < 75 nM [142]. Irreversible changes induced by insufficient vitamin D exposure during early gestation—and prior to the initiation of the supplementation—may explain the lack of effect among women with lower 25OHD levels. It also suggests that maternal 25OHD levels ≥ 75 nM may be required for optimal in utero lung and immune development. Recent follow-up studies of VDAART [143] and COPSAC_2010_ [144] failed to show an effect of prenatal vitamin D supplementation on the development of asthma or recurrent wheeze through to the age of 6 years. Potential explanations for these null results include: The lack of postnatal supplementation in children that failed to sustain the effects of the prenatal supplementation and the effects of vitamin D on virus-induced wheezing and asthma exacerbations in early life that may have prevented some asthma phenotypes from persisting into mid-childhood. Taken together, the results from these two trials suggest that prenatal vitamin D supplementation may be effective in preventing wheezing that occurs in preschoolers, but the effect tends to disappear with time. Preschool wheezing is thought to differ phenotypically from school-aged wheezing in that it is triggered by viral respiratory infections and is transient, with manifestations often regressing by school years. In contrast, school-aged asthma is a more persistent state generally related to the presence of allergies and characterized by an uncontrolled type 2 inflammation. Through its antiviral, antimicrobial, and anti-inflammatory actions, it is plausible that vitamin D may optimize the body’s response to viral infections and hence be more beneficial at an early age. In support of this assumption, a VDAART ancillary study showed that cord blood mononuclear cells (CBMCs) from women allocated to the intervention group exhibited an enhanced proinflammatory cytokine response to innate and mitogenic stimuli [145]. It was hypothesized that high-dose vitamin D enhanced the neonate immune system fitness, thereby preventing asthma development later in life. While in stark contradiction with the findings from in vitro studies using exogenous 1,25(OH)_2_D_3_ [146,147,148], these findings underscore the importance of the timing of the intervention with regards to the effects of vitamin D on the pulmonary and immune systems.

A major limitation of these prenatal trials is that supplementation was not sustained in children after birth. One postnatal vitamin D supplementation trial was undertaken to determine the effect of early vitamin D supplementation in infants particularly at risk of wheezing and vitamin D deficiency. The Wheezing in Black Preterm Infants: Impact of Vitamin D Supplementation Strategy (D-Wheeze) examined the impact of two vitamin D supplementation strategies on the risk of recurrent wheezing by 12 months of age in black preterm infants [149]. As per current recommendations, infants were given a 400 IU vitamin D_3_ supplementation after birth when oral feedings were tolerated. When infants achieved an intake > 200 IU vitamin D from oral feedings of fortified human milk or formulas, they were randomized to either the sustained daily supplementation of 400 IU vitamin D_3_ or a diet-limited supplementation consisting solely of the amount of vitamin D consumed from formula or human milk feedings (placebo wing) until 6 months ‘adjusted age. Results showed a reduction in the risk of developing recurrent wheezing by 12 months’ adjusted age in the sustained supplemented infants (RR 0.66, 95%-CI 0.47–0.94). The design of future trials should combine prenatal and sustained postnatal vitamin D supplementation to provide additional support for the efficacy of vitamin D in the primary prevention of wheezing and asthma. 

In contrast to healthy children, vitamin D insufficiency is more prevalent among children with asthma, both preschoolers [150,151] and older children [152,153]. Cross-sectional studies showed that serum 25OHD is inversely associated with asthma-related outcomes (i.e., frequency of acute attacks and wheeze periods, requirement for high-dose oral corticosteroids) [153,154,155,156,157,158]. Such associations are particularly robust and have been replicated multiple times in various populations [153,155,159,160,161,162,163,164,165]. However, causal inferences are limited by the possibility of reverse causality, where asthma is a cause—rather than a consequence—of the reduced serum 25OHD. This notion is supported by MR studies, which failed to demonstrate any significant association between genetically determined 25OHD levels and asthma [166,167]. In addition, asthmatic children may spend less amounts of time outside, limiting the skin production of vitamin D. Another contributing factor is the widespread misconception that milk and dairy products promote mucus formation, increase airway resistance, and trigger or worsen asthma symptoms [168]. While this belief is not strongly supported by scientific evidence [169,170], it may contribute to the reduced vitamin D intake of these children, although this has never been formally documented. Lastly, asthma is a chronic inflammatory condition and inflammation was postulated to decrease 25OHD levels in other settings [171]. While the impact of inflammation on the vitamin D status of asthmatics remains to be proven, recent data showing dysregulated vitamin D metabolism in adults with asthma, both at baseline and following an intermittent bolus supplementation, provide some support for this hypothesis [172].

The strongest evidence for vitamin D in the secondary prevention of asthma comes from pediatric trials, mostly conducted in schoolchildren. The only vitamin D trials in preschoolers are two pilot trials of 22 and 47 preschoolers aimed at identifying the best supplementation strategy but that were not powered to detect differences in health outcomes [150,151]. A Cochrane systematic review of nine asthma trials (two adult/seven pediatric), with variable use of inhaled corticosteroids, reported a significant protective effect of vitamin D against asthma exacerbations (OR 0.64, 95%-CI 0.46–0.90; 680 participants), the major cause of asthma morbidity and mortality [173]. This protective effect was later confirmed in a meta-analysis of individual patient data performed by the same group. However, the treatment effect failed to reach significance when only pediatric data were included in the analyses [174]. Given that asthma exacerbations are often precipitated by viral respiratory infections for which vitamin D was also found to reduce the incidence of [33,34], it is speculated that vitamin D acts either by preventing the infection or limiting host immune responses that may trigger exacerbations. For example, in rhinovirus and respiratory syncytial virus-infected respiratory epithelial cells, 1,25(OH)_2_D_3_ increased the secretion of AMP cathelicidin [30,175]. It dampened the antiviral and inflammatory response to viruses through the downregulation of nuclear factor NFκB signaling and reduced secretion of antiviral cytokines (e.g., interferon-β (IFN-β)) and chemokines (e.g., IP-10) without, however, compromising viral clearance [176,177]. Vitamin D also downregulated the expression of the intercellular adhesion molecule 1 (ICAM-1) and platelet-activating factor receptor (PAFR), two proteins involved in the adhesion of viruses to the surface of epithelial cells [176]. Through effects on the adaptive immune system, it is also plausible that vitamin D may limit allergen-induced type 2 airway inflammation, which is more associated to school-aged asthma [134]. Vitamin D was shown to increase the expression of the IL-33 decoy receptor ST2, which by binding IL-33, inhibits its capacity to induce the production of Th2 proinflammatory cytokines [178].

In children with moderate or severe exacerbations, daily inhaled corticosteroids are generally indicated, but oral corticosteroids may be required to treat asthma exacerbations. Chronic use of moderate (or higher)-dose corticosteroids in children has detrimental effects on bone health [179] and may slow down growth velocity [180]. Furthermore, viral-induced exacerbations are associated with a poorer response to corticosteroids and increased emergency department management failure [181]. Epidemiologic studies showed an inverse association between low serum 25OHD and the use of more anti-inflammatory medication in asthmatic children [155,160], the need for greater inhaled corticosteroid doses, and an apparent reduction of corticosteroid efficacy [155,182]. Corticosteroids interact with cytosolic receptors (GCR-α), inducing their nuclear translocation and the binding to glucocorticoid response elements located on the promoter of genes involved in anti-inflammatory responses. Corticosteroids induce the expression of MAPK-phosphatase 1 (MKP-1) while repressing that of proinflammatory cytokines tumor necrosis factor (TNF) and IL-8. Vitamin D enhanced the anti-inflammatory effects of corticosteroids by increasing IL-10 secretion by Tregs and MKP-1 expression by mononuclear cells [183]. In addition, vitamin D synergized with corticosteroids to induce a tolerogenic dendritic cell phenotype [184]. A short course of 1,25(OH)_2_D_3_ (0.25 µg twice/day × 4 weeks) given to adults with steroid-resistant asthma improved the clinical response to corticosteroids [185,186]. They showed in vitro that peripheral blood mononuclear cells from steroid-resistant asthmatics given 1,25(OH)_2_D_3_ demonstrated abrogation of dexamethasone-induced IL-17A production and restoration of dexamethasone-induced IL-10 secretion, indicating an overall better corticosteroid responsiveness [185]. Most experimental data, however, come from cell culture experiments designed to obtain maximal effects of pharmacologic dose of 1,25(OH)_2_D_3_ over short time periods and from small trials in adults with steroid-resistant asthma. The implication of vitamin D in corticosteroid responsiveness requires further investigation in pediatric asthma.

While trials in pediatric and adult asthma suggest an effect of vitamin D in reducing the risk of URTIs and asthma exacerbations, the magnitude of effects appears greater in those with the lowest baseline vitamin D status [174]. As such, RCTs are ongoing to address some unresolved issues. For instance, The Vit-D-Kids Asthma study (ClinicalTrials.gov ID: NCT02687815) is designed to assess whether supplementation (4000 IU/day) prevents severe asthma attacks in children with serum 25OHD levels below 30 ng/mL, and who are being treated with inhaled corticosteroids. The DIVA trial (Vitamin D intervention in preschoolers with viral-induced asthma; ClinicalTrials.gov ID: NCT03365687) aims to study the impact of the administration of two boluses of 100,000 IU vitamin D_3_ 3.5 months apart and daily 400 IU D_3_ on the number of asthma exacerbations requiring rescue oral corticosteroids in preschoolers, a high-morbidity population. The results of these trials are expected in the coming years.

### 7.2. Other Allergic Conditions

#### 7.2.1. Atopic Dermatitis

Atopic dermatitis (AD) is a chronic inflammatory skin condition affecting 10% to 20% of children and usually developing within the first year of life. The pathophysiology involves defects in the skin epithelial barrier and deregulated innate and adaptive immune responses, which manifest clinically as relapsing eczematous lesions and pruritus. Compromised barrier integrity facilitates the penetration of allergens and pathogens, which activate keratinocytes and dendritic cells through innate immune receptor ligation by damage-associated molecular patterns (DAMPs) and PAMPs. Activated dendritic cells promote exaggerated Th2-mediated cytokine secretion and inflammation, which further damages the epidermal barrier integrity and increases the propensity to secondary infections [187]. The increased susceptibility of individuals with AD to infections has been linked to a defective capacity to properly increase the production of antimicrobial peptides like cathelicidin following skin lesions [188,189].

In theory, vitamin D should attenuate the chronic immune activation and inflammation seen in AD, yet mechanistic evidence is limited. While keratinocytes are well-known for their involvement in cutaneous synthesis of vitamin D_3_, they also express the VDR and CYP27B1, the expression of which was upregulated in keratinocytes surrounding wounds [190]. The resulting production of 1,25(OH)_2_D_3_ increased the expression of cathelicidin, CD14, and TLR2, further amplifying pathogen recognition and the antimicrobial response during skin repair, hence protecting wounds against infections. Findings from clinical studies tend to support these experimental observations. A three-week vitamin D_3_ supplementation (i.e., 4000IU/day) to 14 individuals with AD (age not specified) led to a marked increase in cathelicidin mRNA levels of lesional skin biopsies whereas the effect of supplementation was non-significant in non-lesional AD biopsies and in biopsies from non-AD controls [191]. In an audit of clinical practice, Albenali et al. assessed CAMP/LL37 peptide levels of lesional and non-lesional skin brushings from vitamin D-insufficient children (i.e., 25OHD < 75 nM) with AD or AD complicated by eczema herpeticum (ADEH), a more severe clinical manifestation [192]. LL-37 levels were measured at baseline and following a 2-month supplementation with daily 6000 IU (1–12 years of age) or 12,000 IU vitamin D_3_ (12–18 years of age), as recommended by the local guidelines for children with 25OHD levels < 50 nM. Children with suboptimal 25OHD levels (50–75 nM) received over-the-counter supplements containing 100% of the recommended daily allowance (RDA) for vitamin D (e.g., 400 IU). At baseline, skin cell levels of LL-37 were associated with serum 25OHD levels and were lower in children with the most severe disease. After 2 months of either therapeutic or over-the-counter vitamin D supplementation, scores of AD severity were markedly reduced whereas LL-37 levels increased by 4-fold and were inversely correlated to AD severity [192]. Other suggested mechanisms of action of the vitamin include downregulation of the monocyte TLR expression, an inhibitory effect on dendritic cells’ activation and Th1 cytokine release, and a reduction of the effect on B cell function, leading to decreased IgE secretion, although no difference was seen between pre- and post-vitamin D supplementation IgE levels in the Albenali study [192]. This finding is consistent with the results of Hyppönen et al. showing a U-shaped relationship between IgE and 25OHD levels in an adult cohort [131]. Similarly, a non-linear relationship was found between cord blood 25OHD levels and the risk of atopy in early childhood [130]. Cord blood 25OHD levels below (<50 nM) and above (>100 nM) a certain level were associated with an increased risk of having detectable inhalant-specific IgE in the first five years of life. It was therefore hypothesized that the maintenance of 25OHD levels between 50 and 100 nM during the first years of life will prevent the development of atopy. 

A recent meta-analysis conducted on observational and intervention studies revealed lower 25OHD levels by 16 nM (95%-CI −31 to −1; *p* = 0.05) in AD vs. non-AD children [193]. This difference was less pronounced in adult studies, where a non-significant difference of 2 nM (95%-CI −5 to 1; *p* = 0.15) was observed, although the limited adult data preclude any firm conclusions. The reasons underlying the striking difference between pediatric and adult data are poorly understood but may relate to sun avoidance habits and low vitamin D intake, as children with AD often suffer from concomitant food allergies and dietary restriction. Mendelian randomization (MR) studies provide evidence that the relationship between 25OHD levels and the risk of atopic diseases is strongly confounded by lifestyle factors [167]. As such, efforts to increase 25OHD levels may likely fail to decrease the risk of developing these conditions. By only addressing the association between genetic determinants of circulating 25OHD levels and disease risk, MR studies overlook the actions of 25OHD and 1,25(OH)_2_D_3_ at the cellular level, which may be beneficial in atopic diseases, but weakly correlated to serum 25OHD. 

All vitamin D trials included in the Hattangdi-Haridas meta-analysis (*n* = 5; 1 adult, 2 pediatric, and 2 mixed) showed improvement in AD severity post-vitamin D oral supplementation [193]. Dosages ranging from daily 1000 to 2000 IU vitamin D for 1 to 3 months led to a reduction in the SCORing Atopic Dermatitis (SCORAD) index of 11 (95%-CI −13 to −9; *p* < 0.001; 3 RCTs) and 21 (95%-CI −27 to −15; *p* < 0.001; 2 studies with repeated measures design) [193]. Of note, all trials included individuals with AD of mild to moderate severity and 25OHD levels below 50 nM. Interestingly, the magnitude of effect exceeds the minimal clinical important difference (MCID) in the SCORAD score previously established for the treatment of AD (i.e., score reduction of 9 points) [194], underscoring the clinical relevance of these observations. Collectively, the results of this meta-analysis provide evidence that vitamin D supplementation of individuals with mild to moderate AD and insufficient vitamin D status leads to clinical improvements in AD severity. 

#### 7.2.2. Food Allergies

Foods do not normally induce allergic reactions due to the ability of the immune system to discriminate between non-self-innocuous and threatening antigens. However, in some susceptible individuals, food antigens trigger inappropriate immune reactions that are mediated, but not always, by antigen-specific IgE. While first exposure to food allergens induces sensitization, their subsequent ingestion results in a larger and faster production of the antigen-specific IgE, triggering the release of mediators, such as histamine, from mast cells. These mediators are responsible for the cutaneous, gastrointestinal, respiratory, and systemic allergic manifestations. Vitamin D was shown to reduce B-cell function, leading to decrease IgE levels in vitro [195]; however, such an observation has been challenged by studies on mice with allergic airway disease in which 1,25(OH)_2_D_3_ administration reduced eosinophilia but resulted in increased serum IgE levels [196]. In contrast, using a different allergy mouse model, Hartmann et al. showed that 1,25(OH)_2_D_3_ significantly reduced total and antigen-specific IgE levels [197], raising the possibility that vitamin D may exert deleterious and beneficial influences depending on the allergic context. Other mechanisms by which vitamin D may modulate the host response to food allergens are the regulation of intestinal tight junctions and innate epithelial defenses, including the antimicrobial capacity, which in turn may influence the composition of the gut microbiota. A reduced diversity of early life gut microbiota has been linked to the onset of allergic diseases [198] whereas cord blood 25OHD levels have been associated with infant gut microbiota, although vitamin D supplementation after birth exerted no effect [199]. Studies specifically addressing the relationship between the vitamin D status, composition of gut microbiota, and allergic diseases are likely to emerge in a near future.

Data from the National Health and Nutrition Examination Survey 2005–2006 (NHANES) revealed an inverse association between 25OHD levels and the risk of aero- and food allergen sensitization for 11 of the 17 allergens tested [200]. Notably, this association was observed in children and adolescents but was not significant in adults. The authors explained this observation by the much shorter time gap between the assessment of vitamin D status and allergic sensitization in children. As most allergies start during childhood, the 25OHD levels measured in adults may not have been reflective of the vitamin D status at the time of the onset of the allergy [200]. In the Australian Health Nuts study, infants with vitamin D insufficiency were three times more at risk of having a food allergy (OR 3.08, 95%CI 1.10–8.59; *p* = 0.032). This association was particularly stronger with peanut (OR 11.51, 95%-CI 2.01–65.79; *p* = 0.006) and multiple food allergies (OR 10.48, 95%-CI 1.60–68.61; *p* = 0.014), two conditions associated with a life-long persistence [201]. The fact that parents were unaware of the food allergy status of their children at the time of enrollment minimized the risk that changes in dietary intake and behavior may have contributed to lowering the vitamin D status prior to study participation. Interestingly, these associations were only found in infants from Australian-born parents, suggesting an unmeasured confounding by genetic, epigenetic, and environmental factors. It is worth noting that, unlike other countries from North America and Europe, Australia does not routinely fortify foods with vitamin D, including cow’s milk. Those results have been replicated in North American cohorts [200,202] but not consistently [203,204], whereas some studies from the German Lifestyle and environmental factors and their Influence on Newborns Allergy risk (LINA) mother-child cohort rather reported positive associations between maternal or cord blood levels of 25OHD and the risk of food allergy [205,206]. 

The premise that early life vitamin D insufficiency may lead to immune deviation and promote the development of allergic diseases has driven investigators to undertake vitamin D supplementation trials targeting infants. In the ongoing VITALITY study, fully breastfed Australian infants aged 6–8 weeks, not receiving any vitamin D supplementation, are allocated either to a placebo or vitamin D arm (daily 400 IU vitamin D_3_) for 12 months (Clinicaltrials.gov ID: NCT02112734) [207]. The main outcome is the prevalence of challenge-proven food allergy at 1 year of age whereas secondary outcomes include the rate of low respiratory tract infections, food sensitization, physician-diagnosed asthma, and vitamin D deficiency. The study is expected to be completed in 2022.

#### 7.2.3. Allergic Rhinitis and Aeroallergen Sensitization

Allergic rhinitis (AR) is a chronic airway inflammatory condition affecting approximately 40% of children that is caused by an IgE-mediated allergic reaction to airborne particles. A meta-analysis of 21 observational studies examining the association between vitamin D status and risk of aeroallergen sensitization or AR showed decreased odds of developing aeroallergen sensitization in children with 25OHD levels ≥ 75 nM vs. < 50 nM (OR 0.54, 95%-CI 0.43 to 0.58; *p* < 0.001) [208]. Strikingly, this result contrasts with that observed in adult studies where a positive association was observed between serum 25OHD and aeroallergen sensitization. The authors explained this unexpected association by the age specificity of immune responses in response to environmental cues. The authors also raised the possibility that the expression of the VDR and enzymes involved in the vitamin D pathway might be age dependent, underlining that the timing of exposure to vitamin D may be a critical determinant to its immune effects. 

One vitamin D trial examined whether vitamin D supplementation during the third trimester of pregnancy and infancy (0 to 6 months) affects aeroallergen sensitization [209]. Mothers and infants were randomized to either a placebo, a low- (1000 IU/day in mother from 27 weeks of gestation to child’s birth and 400 IU daily to infants until 6 months of age), or a high-vitamin D_3_ group (2000 IU/day to mothers and 800 IU daily to infants, same durations). Aeroallergen sensitization and acute primary healthcare visits due to respiratory illnesses were assessed in children at 18 months of age. Risk of house dust mite sensitization was significantly reduced in children from the high-dose vitamin D group compared to the placebo (RR = 0.34, 95%-CI 0.12–0.94). As for primary care visits for respiratory illnesses, group differences were seen for asthma, where the proportion of children with healthcare visits in which asthma was recorded as being present was significantly higher in children from the placebo group [209]. Interestingly, these findings add support to the positive effect of pre- and postnatal vitamin D supplementation on asthma development during childhood. 

## 8. Conclusions

Vitamin D deficiency and rickets in several populations remains an under-recognized clinical problem, and its significance extends beyond skeletal health to non-classical actions of vitamin D, including a range of immune-related diseases. There is solid evidence that vitamin D supplementation can reduce the rates of infections in pediatric populations. There is also growing evidence for a beneficial role of supplementation in preventing autoimmune disorders, and there is promising data linking vitamin D deficiency to increased rates of childhood asthma and other allergic conditions. The use of vitamin D in the primary prevention of asthma needs to be supported by trials including the supplementation of mothers throughout pregnancy and of their children postnatally. With regard to the potential of vitamin D in improving asthma and other allergic conditions’ management, there remains a need for large-scale RCTs to confirm many of these findings.

## Figures and Tables

**Table 1:** Unpublished randomized controlled trials on the effect of vitamin D in pediatric immune conditions.

Clinicaltrials.gov Identifier	Setting	*n* *	Population	Interventions	Primary Outcomes	Expected Completion Year
**Crohn’s Disease**						
NCT03999580 (ViDiPeC-2)	Canada	316	Pediatric Crohn’s disease patients (4–18 years)	3000 or 4000 IU/day, acc. to body weight for 4 weeks, 2000 IU/day for 48 weeks. Control: 600 IU/day induction (4 weeks) and maintenance (48 weeks).	Number of relapses and quality of life, incl. levels of physical activity	2024
**Asthma**						
NCT03365687 (DIVA)	Canada	864	Preschoolers (1–5 years) with viral infection-triggered wheezing/asthma	Two oral boluses of 100,000 IU vitamin D_3_ 3.5 months apart with daily 400 IU vitamin D_3_ for 7 months. Placebo boluses, daily placebo	Number of asthma exacerbations treated with rescue OCS	2023
NCT02687815 (Vit-D-Kids Asthma)	USA	400	Schoolchildren (6–16 years) Treated with ICS Vitamin D insufficient (25OHD < 75 nM)	Daily 4000 IU vitamin D_3_ or placebo for 48 weeks	Severe asthma exacerbations requiring systemic CS or an increase in stable maintenance dose for at least 3 days Asthma-related hospitalization or emergency room visit requiring OCS	Completed, unpublished
**Food allergies**						
NCT02112734 (Vitality)	Australia	3555	Healthy term infants (6 to 12 weeks) Predominantly breastfed	Daily 400 IU vitamin D_3_ or placebo for 12 months	Prevalence of challenge-proven food allergy at 1 year of age	2022

* Estimated sample size; OCS = oral corticosteroids; ICS = inhaled corticosteroids.
